# Effects of selection for cooperation and attention in dogs

**DOI:** 10.1186/1744-9081-5-31

**Published:** 2009-07-24

**Authors:** Márta Gácsi, Paul McGreevy, Edina Kara, Ádám Miklósi

**Affiliations:** 1 Dept. of Ethology, Eötvös University, H-1117, Budapest, Pázmány P. s. 1/c., Hungary; 2 Faculty of Veterinary Science, University of Sydney, NSW 2006, Australia

## Abstract

**Background:**

It has been suggested that the functional similarities in the socio-cognitive behaviour of dogs and humans emerged as a consequence of comparable environmental selection pressures. Here we use a novel approach to account for the facilitating effect of domestication in dogs and reveal that selection for two factors under genetic influence (visual cooperation and focused attention) may have led independently to increased comprehension of human communicational cues.

**Method:**

In Study 1, we observed the performance of three groups of dogs in utilizing the human pointing gesture in a two-way object choice test. We compared breeds selected to work while visually separated from human partners (N = 30, 21 breeds, clustered as independent worker group), with those selected to work in close cooperation and continuous visual contact with human partners (N = 30, 22 breeds, clustered as cooperative worker group), and with a group of mongrels (N = 30).

Secondly, it has been reported that, in dogs, selective breeding to produce an abnormal shortening of the skull is associated with a more pronounced area centralis (location of greatest visual acuity). In Study 2, breeds with high cephalic index and more frontally placed eyes (brachycephalic breeds, N = 25, 14 breeds) were compared with breeds with low cephalic index and laterally placed eyes (dolichocephalic breeds, N = 25, 14 breeds).

**Results:**

In Study 1, cooperative workers were significantly more successful in utilizing the human pointing gesture than both the independent workers and the mongrels.

In study 2, we found that brachycephalic dogs performed significantly better than dolichocephalic breeds.

**Discussion:**

After controlling for environmental factors, we have provided evidence that at least two independent phenotypic traits with certain genetic variability affect the ability of dogs to rely on human visual cues. This finding should caution researchers against making simple generalizations about the effects of domestication and on dog-wolf differences in the utilization of human visual signals.

## Background

It has been suggested that the study of the domestic dog might help to explain the evolution of human communicative skills, because the dog has been selected for living in a human environment and engaging in communicative interactions with humans for more than 10,000 years [1,2]. More specifically, it was assumed that the functional similarities in the socio-cognitive behaviour of dogs and humans emerged as a consequence of comparable environmental selection pressures. Earlier it was thought that selection might have acted directly on the cognitive capacity of dogs [3]. However, subsequent studies have emphasized the role of auxiliary components of behaviour, changes in which may have facilitated the manifestation of the pre-existing cognitive abilities in an anthropogenic environment [see "emotional reactivity" hypothesis in [1]]. Indeed, any performance in a cognitively challenging task does not depend only on mental machinery, but on other factors such as temperament. Recently, considerable attention has been paid to the so-called human-cued "two-way object choice" test, in which the subject can capitalize on the gestural cue of a human to choose between two containers [see [2], for a review]. The relatively high performance of dogs in this task in comparison with apes [3] and wolves [4,5] was explained chiefly by reference to selective factors that acted directly on the cognitive ability of dogs to respond to human gestural cues. Recent findings on adult wolves' higher performance were attributed either to learning effects related directly to the task [6] or to developmental changes in some behavioural traits [7].

Importantly, however, there are at least two other situational factors that affect the performance of the subjects in this task. Firstly, Hare [8] argued that the versions of the two-way choice task, where the subject has to select the social signal in preference to other cues (e.g. the location of the bait in the previous trial), are cooperative in nature. The poor performance of the chimpanzees in this test was attributed to their putative inability to appreciate the cooperative aspect of this task. Secondly, performance may be affected by enduring attention, i.e., that watching the cueing human more intensely or for longer could also facilitate performance because it increases the subjects' probability of noticing and correctly recognizing minute gestural signals. Differences in attention span appear critical in social learning [9] where the quality of observation also affects subsequent performance. Previously we have shown that an increased tendency to look at humans may also play role in dog-wolves differences [4,10].

Recent data on the performance of dogs and wolves in the comprehension of the distal pointing gestures suggest that claims about dogs' general superior ability in relying on subtle human communicative signals remain largely debatable [7]. This is partly because individuals from some dog populations do not show high levels of performance [6]. Recent reports and resultant discourse [see [3,4,6]] have emphasized the possibility that the comparisons of dog and wolf groups have failed to exclude the influence of confounding environmental effects.

Without relying on species comparisons, in the current two studies we offer a novel approach to reveal the possible effects of artificial selection of dogs; selection that might affect their performance in communicative tasks. In the two-way object choice test, we applied the relatively demanding distal momentary pointing as a human cue, because it has been established as a benchmark by several previous studies [e.g. [5,11]; 11; for review see [2]]

Our basic assumption is that, during domestication, dogs have been selected for 1) enhanced cooperative ability and 2) enduring attention, and these skills have been further differentiated during the process of breed formation.

Investigation of the working history of different breeds suggests that dogs have been required to cooperate in two fundamentally different contexts. Some work cooperatively, with continuous visual contact of their human partner, (e.g. herding dogs, gundogs), labelled the 'cooperative worker' breeds, whereas others work with no human visual contact (e.g. hounds, earth dogs, livestock guarding dogs and sled dogs), labelled the 'independent worker' breeds. Since the human cueing is necessary for the success in the two-way choice task, we firstly hypothesized that dogs selected for cooperation in visually guided tasks would show superior performance in comparison with the other working breeds.

Our second hypothesis emerged from a recent study that detected a significant difference in the distribution of retinal ganglion cells between brachycephalic ("short-nosed") and dolichocephalic ("long-nosed") dog breeds [12]. It reported that ganglion cells in brachycephalic breeds occur more centrally in the retina. Since, in other species, such arrangement usually correlates with the retinal location of greatest visual acuity, McGreevy et al. [12] suggested that brachycephalic breeds might respond most to stimuli in the central field (i.e., when looking forward) because they are less disturbed by visual stimuli from the peripheral field. Accordingly, we hypothesized that this morphological change could also influence performance in the two-way choice task and gave brachycephalic breeds an advantage over dolichocephalic breeds.

## Method

Using a large sample of subjects (n = 180), Gácsi and colleagues [13] have already showed that the performance of more than two-month-old pet dogs does not depend on age or sex in the two-way object choice test. Moreover, even such major environmental factors as living in the house versus the garden, training for special skills (agility) and the amount of active daily interaction with the owner do not influence success in this task. Nevertheless, the current study used age- and sex-matched samples that were balanced in several respects in order to avoid potential associations with different ways of handling in the groups. The samples in both studies were balanced for the duration of daily interaction, type of training (grouping the dogs into three categories: no training; basic obedience; special courses), management conditions (in or outside the house), and the age at which the dogs had been obtained.

### Subjects

A total of 140 pet dogs were tested in these two studies. Subjects were recruited from dog schools, and from our Family Dog Project volunteer database. All subjects were socialized about at the same level in human families, none of them were chained or kept in kennel, and all were walked regularly and/or attended a puppy class or basic obedience courses at a dog school.

The protocol was the same as in the developmental study of dogs by Gácsi and co-workers [13]. Most of the tests were video recorded but, in some cases, the choices of the dogs were recorded only by hand-written notes.

In Study 1, three groups of dogs were tested. The independent worker group consisted of breeds selected to work while visually separated from human partners. These include hounds, earth dogs (dogs used for underground hunting), livestock guard dogs, sled dogs (N = 30 from 21 breeds, 14 males and 16 females, mean age was 2.66 years). The cooperative worker group, involved breeds selected to work while in continuous visual contact with human partners (sheepdogs, gundogs; N = 30 from 22 breeds, 14 males and 16 females, mean age was 2.71 years). (Table 1)

**Table 1:** Data and results of individuals in the independent and cooperative worker groups

**Independent worker breeds**				**Cooperative worker breeds**			
	**breed**	**subgroup**	**correct choice**		**breed**	**subgroup**	**correct choice**

1	Caucasian ovcharka	guard	12	1	Australian shepherd	sheepdog	19

2	Great Pyreneen	guard	9	2	Border collie	sheepdog	12

3	Komondor	guard	16	3	Border collie	sheepdog	17

4	Komondor	guard	9	4	Briard	sheepdog	19

5	Kuvasz	guard	12	5	Dutch shepherd	sheepdog	9

6	Bedlington terrier	earth	9	6	German shepherd	sheepdog	17

7	Cairn terrier	earth	16	7	German shepherd	sheepdog	13

8	Dachshound	earth	11	8	Groenendale	sheepdog	15

9	Jack Russell	earth	14	9	Kelpie	sheepdog	6

10	Jack Russell	earth	12	10	Malinois	sheepdog	12

11	Parson Russell	earth	11	11	Mudi	sheepdog	12

12	Parson Russell	earth	12	12	Pumi	sheepdog	11

13	Welsh terrier	earth	14	13	Puli	sheepdog	16

14	West H. W. terrier	earth	15	14	Tervueren	sheepdog	18

15	Basset hound	hound	17	15	Tervueren	sheepdog	15

16	Basset hound	hound	16	16	Rough collie	sheepdog	12

17	Beagle	hound	10	17	Shetland sheepdog	sheepdog	10

18	Beagle	hound	11	18	Shetland sheepdog	sheepdog	19

19	Bloodhound	hound	19	19	Tibetan terrier	sheepdog	15

20	Hannover hound	hound	10	20	German pointer	gundog	15

21	Hungarian greyhound	hound	19	21	Golden retriever	gundog	12

22	Hungarian greyhound	hound	12	22	Golden retriever	gundog	20

23	Slovak hound	hound	11	23	Irish setter	gundog	14

24	Transylvanian hound	hound	9	24	Labrador	gundog	18

25	Whippet	hound	10	25	Labrador	gundog	13

26	Alaskan malamute	sled	10	26	Labrador	gundog	12

27	Siberian husky	sled	14	27	Hungarian vizsla	gundog	15

28	Siberian husky	sled	9	28	Hungarian vizsla	gundog	18

29	Siberian husky	sled	9	29	Hungarian vizsla	gundog	13

30	Siberian husky	sled	16	30	Weimaraner	gundog	15

The third group consisted of mongrels (N = 30, 13 males and 17 females, mean age was 2.17 years). Importantly, from this third group we excluded dogs that looked like a purebred but had no pedigree and dogs that were F1 crosses of two breeds.

In Study 2, we tested two groups. One group consisted of individuals from brachycephalic breeds with high cephalic index (skull width/skull length) and more frontally placed eyes (N = 25 from 14 breeds, 16 males and 9 females, mean age was 2.12 years). In the other group, we tested individuals from dolichocephalic breeds with low cephalic index and laterally placed eyes (N = 25 from 14 breeds, 9 males and 16 females, mean age was 2.44 years).

Note, that there were no individuals from the working breeds in brachycephalic group, which made a cross analysis impossible. (Table 2).

**Table 2:** Data and results of individuals in the dolichocephalic and brachycephalic breed groups

**Dolichocephalic breeds**			**Brachycephalic breeds**		
	**breed**	**correct choice**		**breed**	**correct choice**

1	Afghan hound	13	1	American bulldog	19

2	Bedlington terrier	8	2	Boxer	12

3	Bedlington terrier	12	3	Boxer	18

4	Dachshound	16	4	Boxer	13

5	Dachshound	10	5	Bulldog	20

6	Doberman	14	6	Bullmastif	19

7	Doberman	12	7	Bullmastif	14

8	English setter	11	8	Cavalier K. Ch. spaniel	16

9	Foxterrier	12	9	Cavalier K. Ch. spaniel	11

10	Irish setter	14	10	Cavalier K. Ch. spaniel	17

11	Irish setter	11	11	Chow-chow	18

12	Hungarian greyhound	10	12	Dogo Canario	16

13	Hungarian greyhound	16	13	French bulldog	16

14	Hungarian greyhound	14	14	French bulldog	20

15	Podenco Ibicenco	18	15	Newfoundland	16

16	Rough collie	16	16	Pug	20

17	Rough collie	12	17	Pug	19

18	Rough collie	12	18	Pug	10

19	Russian greyhound	11	19	Rottweiler	11

20	Shetland sheepdog	12	20	Rottweiler	16

21	Shetland sheepdog	15	21	Rottweiler	20

22	Shetland sheepdog	11	22	Shar pei	18

23	Welsh terrier	10	23	Shar pei	19

24	Whippet	10	24	Staffordshire bull terrier	12

25	Whippet	12	25	Tibet spaniel	20

### Procedure

The tests took place in an unfamiliar room at two locations: the Department of Ethology, ELTE Budapest, and the Top Mancs Dog Training Centre. Two plastic bowls (measures: 10–25 cm in diameter, 10–25 cm height depending on the size of the dog) were used to hide the bait. We hid small pieces of cold cut as bait. Both bowls were thoroughly scented with the food before the experiment.

In the pretraining, the experimenter (E) placed the two bowls in front of herself, 1.8–2 m apart. She dropped a piece of food into one of the bowls while the subject was held by the owner at a distance of 2–2.5 m from her. As soon as the food had fallen into the bowl, the owner released the subject and it was allowed to eat the food. This procedure was repeated twice for each bowl to ensure that the subject learned that the bowls might contain food. After a short break, the test session began.

During the test, the arrangement of the bowls, the E, the subject and the owner were the same as described above. To prevent the dog from observing the baiting procedure, the E turned away from the subject while she put a piece of food into one of the bowls. The owner made the subject sit or stand facing the E, while the E placed both bowls onto the floor at the same time in front of her. During the pointing, the E stood facing the subject at a distance of 2–2.5 m with her arms folded in front of her chest and established eye contact with the subject prior to signalling. For a few very small dogs (N = 2 in dolichocephalic group and N = 3 in all other groups), the E presented the pointing gesture in a kneeling position (in this case the elbow was pressed to the waist so the distance was the same between the pointing finger and the bowl).

The owner stood behind the dog and held its collar until the E gave the cue. If the subject did not gaze at the E's face, she called it by its name or clapped her hands to attract its attention. As soon as the eye contact was achieved, the E enacted a momentary distal pointing gesture (see also 5, 13). This is a short, definite pointing toward the baited bowl with the outstretched index finger about 60–80 cm from the bowl. The E's arm was in pointing position for less than a second, and then her hand was placed back on her chest. The subject was released only after the hand had returned to its starting position. Throughout the trial, the E was looking at the subject. If the subject did not leave the starting point within 3 sec of being released, the E repeated the pointing gesture. Whichever bowl the subject first approached (within a distance of 10 cm) was considered the chosen bowl. The subject could eat the food only if it chose the baited bowl, otherwise the baited bowl was picked up.

The test session included 20 trials for each subject. In half of the trials, the baited bowl was placed on the right side, in the other half it was on the left. The order of baiting was defined randomly with the restrictions that one side could be rewarded only twice in a row and that two consecutive baitings in the same bowl could not arise at the very beginning of the trial.

### Data analysis

We coded and counted the correct choices of every dog. For group level analyses, we calculated the percent of correct choices from the 20 trials for each individual. In Study 1, one-way ANOVA (with Bonferroni post-hoc test) was used to compare the different groups' performance. In Study 2, independent sample t-test was applied. For all groups, one-sample t-tests were used to compare the results against chance performance (50%). Importantly, the individual performances were also analyzed statistically with binomial tests (according to the binomial distribution, 5 errors out of 20 trials result in a p-value of 0.041, so a subject can be reported as relying on the pointing gesture over chance if it achieved 15 or more correct choices). We applied the Pearson X^2 ^test to compare the rate of the successful individuals in different groups.

## Results

### Study 1 – comparison of working breeds selected for different purposes

The mean performance of all three groups was better than chance (independent worker: t_(29) _= 4.384; p < 0.001, cooperative worker: t_(29) _= 7.219; p < 0.001, mongrel: t_(29) _= 4.785; p < 0.001).

The comparison of the results showed significant difference among the three groups (F_2,87 _= 5.852; p = 0.004). The post-hoc tests revealed better performance of the cooperative worker breeds compared with the independent worker group (p = 0.03) and the mongrels (p = 0.006) as well.

**Figure 1:**
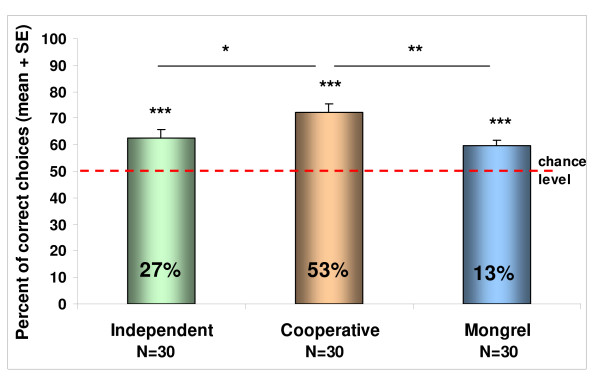
**Performance of the independent and cooperative worker groups and the mongrels in Study 1**. Columns show the group results of independent workers, cooperative workers and mongrels. Stars on top of the columns show significant difference compared to chance level (one sample t-test). Stars above the lines show significant difference between groups (ANOVA). * p < 0.05; ** p < 0.01; *** p < 0.001. The numbers in the columns show the percentage of the successful subjects within a group (measured at the individual level with binomial test).

Comparing the proportion of the successful individuals in the three groups, we found similar differences (X^2^_2 _= 11.61; p = 0.003). There were more successful individuals in the cooperative worker group than in the independent worker (X^2^_1 _= 4.44; p = 0.035), and the mongrel group (X^2^_1 _= 10.8; p = 0.001) (Figure 1).

### Study 2 – comparison of brachycephalic and dolichocephalic breeds

The mean performance of both groups was better than chance (brachycephalic: t_(24) _= 9.798; p < 0.001, dolichocephalic: t_(24) _= 5.204; p < 0.001).

By comparing the mean performance of the two groups, however, we found that the brachycephalic breeds were more successful than the dolichocephalic breeds (t_(48) _= -4.848; p < 0.001).

**Figure 2:**
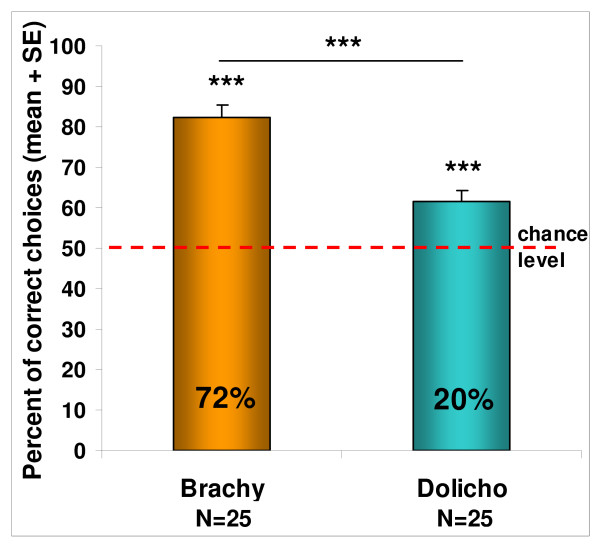
**Performance of the brachycephalic and dolichocephalic breed groups in Study 2**. Columns show the group results compared to chance level (one sample t-test) and to each other (independent sample t-test). *** p < 0.001. The numbers in the columns show the percentage of the successful subjects within a group (measured at the individual level with binomial test).

The comparison of the individual success rates in the two groups also confirmed the group level differences with more successful individuals in the brachycephalic than in the dolichocephalic group (X^2^_1 _= 13.6; p < 0.001). (Figure 2)

## Discussion

Regardless of our categorization, all groups of dogs showed an ability to rely on the human momentary distal pointing. However, the current study is the first, to our knowledge, to reveal striking difference in the performance of breed groups selected for different characteristics.

In accordance with our hypothesis, the performance of working dog breeds selected for intense visual contact with the owner proved to be better in the utilization of the human distal momentary pointing gesture than those selected for working independently of or visually separated from the owner. We suggest that this might not be attributable to differences in the cognitive abilities *per se*, but rather reflects a genetic tendency to be responsive to social stimuli in a cooperative context. Frank [14] proposed that one important difference between dogs and wolves is that dogs can more easily be brought under the control of artificial stimuli. For example, dogs can be trained also to respond to verbal or non-verbal human cues whilst this is usually difficult with wolves. However, in the current study, a general sensitivity gained during the process of domestication does not account for differences in performance of independent and cooperative working dogs. It is more likely that direct selection for utilizing human visual signals endowed cooperative breeds with an ability to inhibit their own spontaneous behaviour and benefit from human social cues. Considering the nature of such visually guided cooperative interactions between dog and human, e.g. herding a flock of sheep, dogs could benefit from being able to change their behaviour from one moment to the next according to human stimuli and to inhibit decisions based exclusively on their own assessment of the current situation. Accordingly, dogs bred to work under continuous visual cuing were probably more inclined to observe the human gestural behaviour, and also were less influenced by other aspects of the experiment, e.g. the location of the reward in the previous trial which can contribute to poorer performances [see [13]].

It is important to stress that all dogs in this study had been socialized in human families as puppies, and the groups were balanced for housing conditions, the time of active interaction between owners and dogs, and also for training. Thus, it seems unlikely that differences (both negative and positive) in experience of communicating with humans could have significantly influenced the results. In this respect, the relatively low performance of mongrels seems somewhat contradictory. Although they found the cued bait significantly more often than expected by chance, especially at an individual level their performance showed little evidence of comprehension. Given that, due to the balancing of the samples, their rearing social environment did not differ from that of subjects in the other groups (most of them lived in the house and had obedience or agility training), we assume that genetic factors may have influenced their performance. Unfortunately, in general very little is known about the population and molecular genetics of mongrels (which are, in our case, not cross-breeds of known purebred parents but rather mainly the descendents of mongrels), so our explanation of the present results is based on a most plausible scenario. Assuming that present day mongrels originate from a population that has been under continuous selection for independent survival skills, because, for example, their reproduction was not supported by humans, then one could assume that independent problem-solving abilities would prevail over the motivation to be guided. This hypothesis would predict superior performance of mongrels (and also independent workers) compared with cooperative worker breeds in tasks requiring independent problem-solving abilities.

In Study 2, as predicted, we found that the brachycephalic breed group was significantly better in using the human pointing gesture than the dolichocephalic group. The superior performance of these dogs can be explained by their much more focused attention on the signaller, and strongly indicate that a difference in a morphological characteristic can also influence the performance in communicative task. These results could also support the suggestion by McGreevy at al (2004) that it might not have been the paedomorphic facial appearance of these dogs, which was selected for, as assumed by Lorenz [15] and many others (referred to as "Kinderschema"). Instead, humans might have preferred animals that looked at them for longer durations because this enhanced the effectiveness of communicative interaction.

The current study explored variance within dog breeds/types or breed groups. As such, it might offer novel account for selective influences on behaviour and performance. In contrast to wolf-dog studies, breed comparisons can be better controlled for a range for environmental variables including socialization and individual experience. Furthermore, any dog-specific comparative studies can now aim at addressing more specific research questions. Finally, our current work could also provide the raw material for genetic studies that use large numbers of subjects to explore components of the genetic mechanisms that influence, for example, head shape or size [e.g. [16,17]]. Similar approaches have been reported recently by Jones et al [18] who identified quantitative trait loci (QTL) on different chromosomes when looking for some phenotypic breed stereotypes in dogs.

## Limitations

Analyzing only success and not behaviours (such as latency of attention or gazing at the correct direction before choosing), may have limited the power to detect important additional differences between groups.

Future comparisons in different communicational paradigms as well as in problem solving contexts may lead to clearer interpretation of the current findings.

Only the two extremities of the dogs in respect of head shape have been tested. The study needs to be replicated on an independent sample balanced for all other potential factors, and it is to be determined, whether there is a correlation between the performance and the head shape.

## Conclusion

After controlling for environmental factors, we have provided evidence that at least two independent phenotypes affect the ability of dogs to rely on subtle human visual cues. This finding should caution researchers against making crude generalizations about the effects of domestication and on dog-wolf differences in the utilization of human visual signals.

## Competing interests

The authors declare that they have no competing interests.

## Authors' contributions

MG coordinated the study, directed the data processing, conducted the statistical analysis, and drafted the manuscript. PM initiated the research study and reviewed drafts. EK aided in the study coordination, obtained questionnaire data from owners, tested dogs, processed and analyzed the data. ÁM conceptualized the study, contributed to the research design, aided in the interpretation of the data, and drafted the manuscript. All authors were involved in the revision of the manuscript and have given final approval of the final manuscript.
